# Data on the effects of Charcot-Marie-Tooth disease type 2N-associated AARS missense mutation (Arg329-to-His) on the cell biological properties

**DOI:** 10.1016/j.dib.2019.104029

**Published:** 2019-05-24

**Authors:** Naoko Imaizumi, Yu Takeuchi, Haruka Hirano, Tomohiro Torii, Yoichi Seki, Takako Morimoto, Yuki Miyamoto, Hiroyuki Sakagami, Junji Yamauchi

**Affiliations:** a Laboratory of Molecular Neuroscience and Neurology, Tokyo University of Pharmacy and Life Sciences, Hachioji, Tokyo 192-0355, Japan; b Department of Neuroscience, Baylor College of Medicine, Houston, TX 77030, USA; c Department of Pharmacology, National Research Institute for Child Health and Development, Setagaya, Tokyo 157-8535, Japan; d Department of Anatomy, Kitasato University School of Medicine, Sagamihara, Kanagawa 252–0734, Japan

**Keywords:** CMT2, CMT2N, AARS, Disease-associated mutation, Cell biological property

## Abstract

Charcot-Marie-Tooth (CMT) diseases are genetic neuropathies in the peripheral nervous system (PNS). Type 1 CMT diseases are neuropathies in Schwann cells, PNS myelinating glial cells, whereas type 2 CMT diseases are axonal neuropathies. In addition, there are other types of categories in CMT diseases. CMT diseases are associated with approximately 100 responsible genes. Taiwanese mutation (Asn71-to-Tyr) of alanyl-tRNA synthetase (AARS) in type 2N CMT disease has been reported to have several pathological effects on properties of AARS proteins themselves [1]. Also, some mutations in other responsible genes affect cell biological properties of their gene products [2,3]. Herein we provide the data regarding the effects of another type 2N CMT disease-associated AARS mutation (Arg329-to-His) in French family on the cellular properties.

**Table:** Specifications Table

Subject area	Biology
More specific subject area	Molecular and cellular neurobiology, Molecular and cellular neurology
Type of data	Figure
How data was acquired	Immunofluorescence, microscopy
Data format	Raw data, analyzed data
Experimental factors	Cell lines were transfected with the plasmid encoding the wild type construct or mutant one and were used for experiments.
Experimental features	Immunofluorescence analysis, cell differentiation analysis
Data source location	Laboratory of Molecular Neuroscience and Neurology, Tokyo University of Pharmacy and Life Sciences, Tokyo, Japan
Data accessibility	Data is available with this article
Related article	Y. Tatsumi, N. Matsumoto, N. Iibe, N. Watanabe, T. Torii, K. Sango, K. Homma, Y. Miyamoto, H. Sakagami, J. Yamauchi, CMT type 2N disease-associated AARS mutant inhibits neurite growth that can be reversed by valproic acid. Neurosci. Res. in press: DOI: 10.1016/j.neures.2018.09.016.

**Table:** 

**Value of the data** • This data set is of value to the scientific community to need the information for the effects of various types of Charcot-Marie-Tooth disease-associated mutations on cell biological changes. • The data can provide the method to examine changes of the properties of gene products by Charcot-Marie-Tooth disease-associated mutations. • The data allow us to promote how various Charcot-Marie-Tooth disease type 2-associated mutations have similar effects *in vitro.*

## 1 Data

**Fig. 1:**
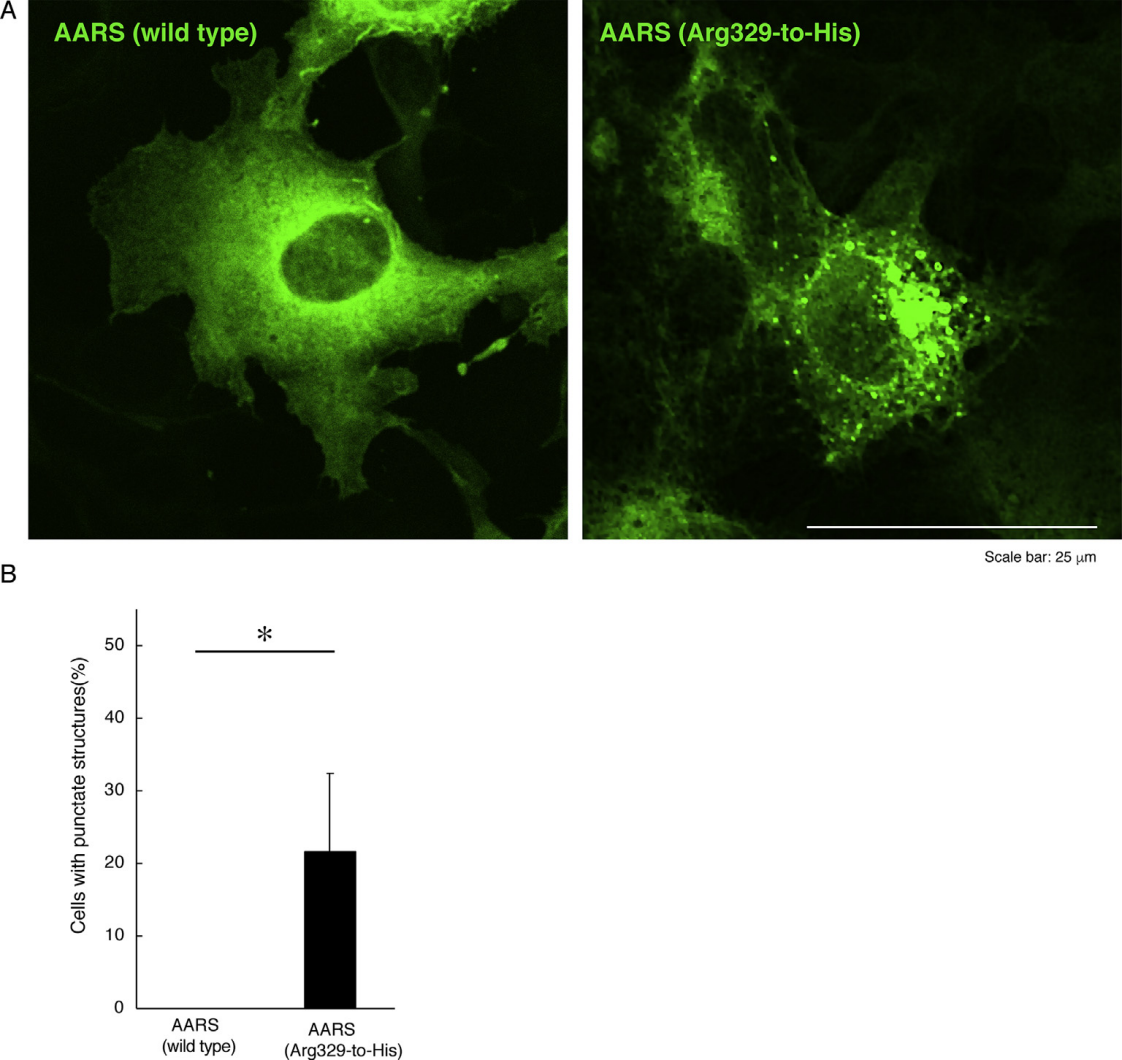
**GFP-tagged wild type AARS is localized in cytoplasm and the mutant proteins are localized in punctate structures.** (A) COS-7 cells were transfected with the plasmid encoding GFP-tagged wild type AARS or the mutant (Arg329-to-His) and their confocal microscopic green fluorescent images were simply captured. Representative cytoplasmic wild type AARS and mutant AARS in punctate structures are shown. (B) Percentages of cells with GFP-tagged proteins exhibiting punctate localization are statistically shown (*, p < 0.01 of Student's t-test; n = 3 experimental fields). In cells expressing GFP-tagged wild type AARS, cells exhibiting the punctate localization were 0 for 31 cells of the total in experiment #1, 0 for 29 cells of the total in experiment #2, and 0 for 19 cells of the total in experiment #3. In cells expressing mutant AARS, cells exhibiting the punctate localization were 9 for 25 cells of the total in experiment #1, 3 for 31 cells of the total in experiment #2, and 4 for 21 cells of the total in experiment #3. Total counted cell numbers were 79 and 77 in cells expressing wild type AARS and the mutant, respectively.

**Fig. 2:**
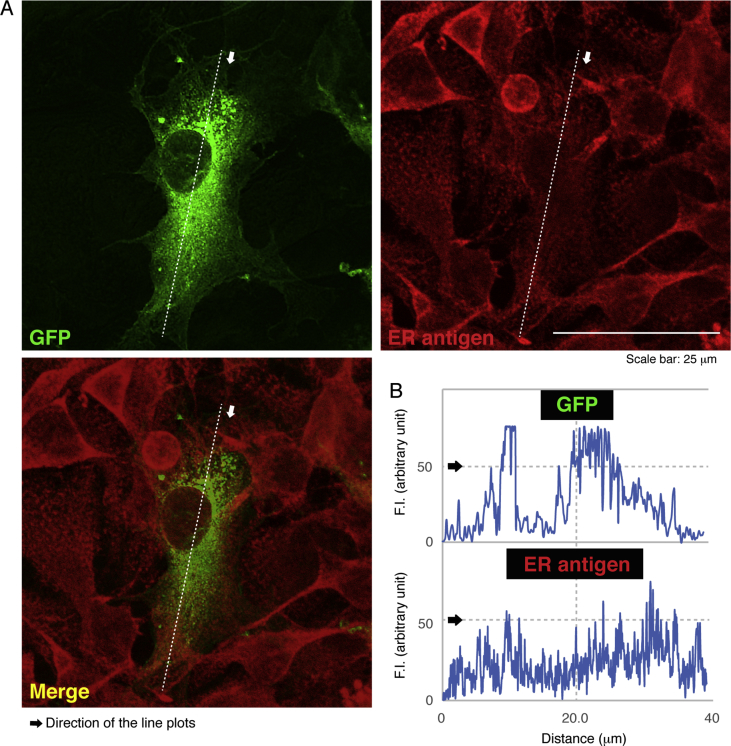
**AARS mutant proteins are not localized in the ER.** (A) Cells were transfected with the plasmids encoding GFP-tagged AARS mutant (green) and stained with an anti-ER antigen antibody (red). Representative green, red, and merged (yellow) images are shown. (B) Scan plots along a white dotted line in the arrow direction were performed, and graphs showing fluorescent intensities (F.I., arbitrary unit) can be seen in the bottom right panels.

**Fig. 3:**
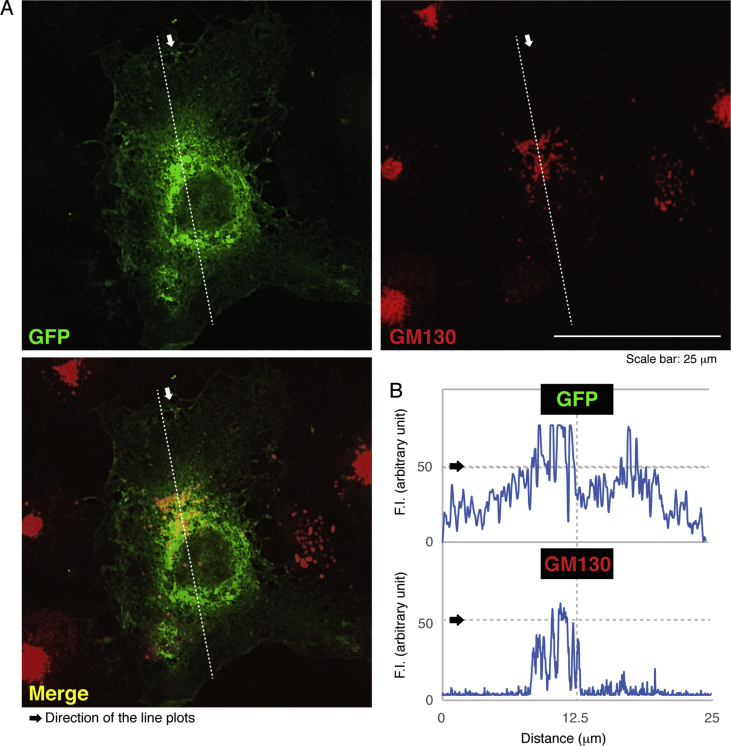
**AARS mutant proteins are partially localized in the Golgi body.** (A) Cells were transfected with the plasmids encoding GFP-tagged AARS mutant (green) and stained with an anti-GM130 antibody (red). Representative green, red, and merged (yellow) images are shown. (B) Scan plots along a white dotted line in the arrow direction were performed, and graphs showing fluorescent intensities (F.I., arbitrary unit) can be seen in the bottom right panels.

**Fig. 4:**
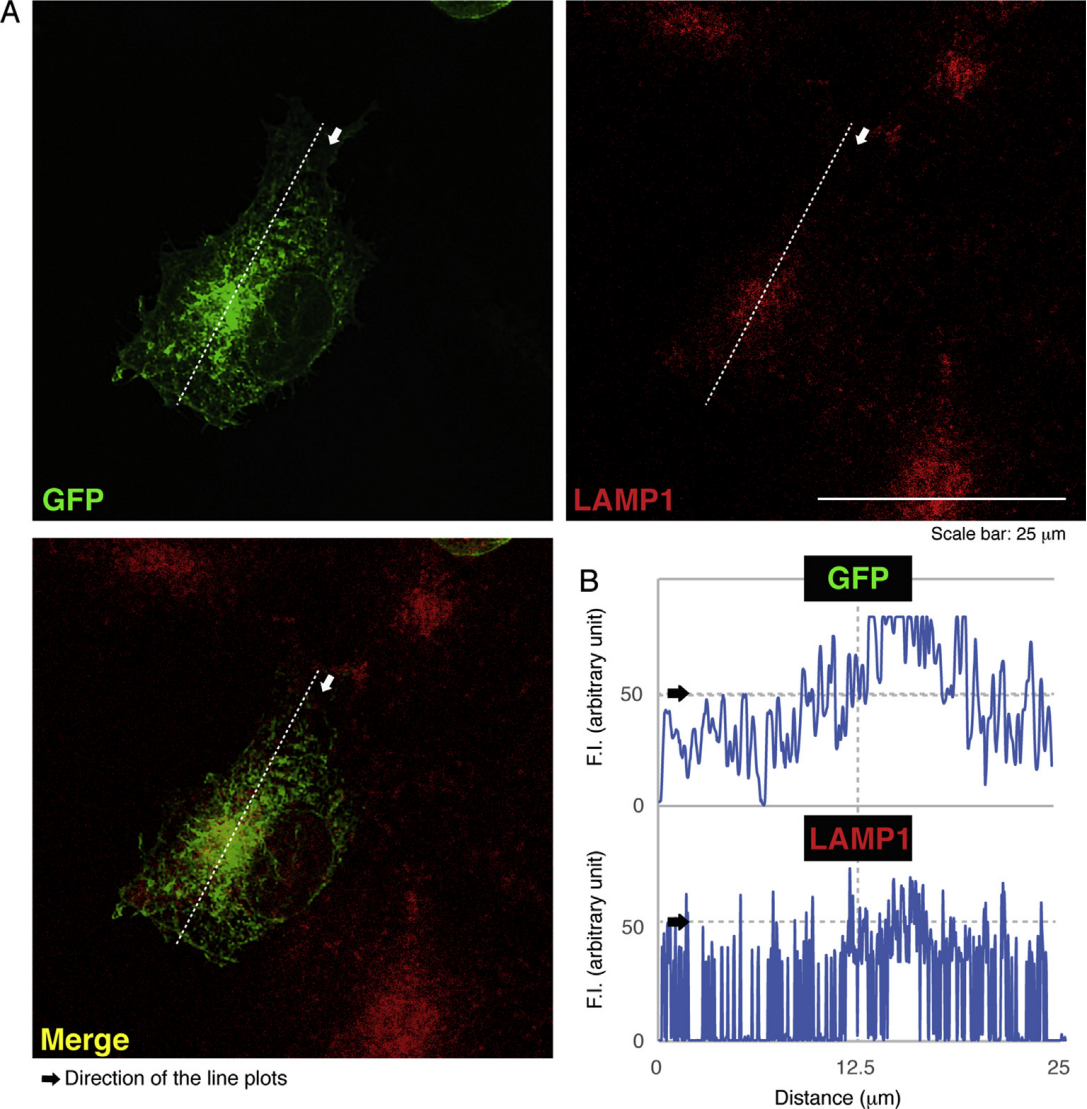
**AARS mutant proteins are partially localized in the lysosome.** (A) Cells were transfected with the plasmids encoding GFP-tagged AARS mutant (green) and stained with an anti-LAMP1 antibody (red). Representative green, red, and merged (yellow) images are shown. (B) Scan plots along a white dotted line in the arrow direction were performed, and graphs showing fluorescent intensities (F.I., arbitrary unit) can be seen in the bottom right panels.

**Fig. 5:**
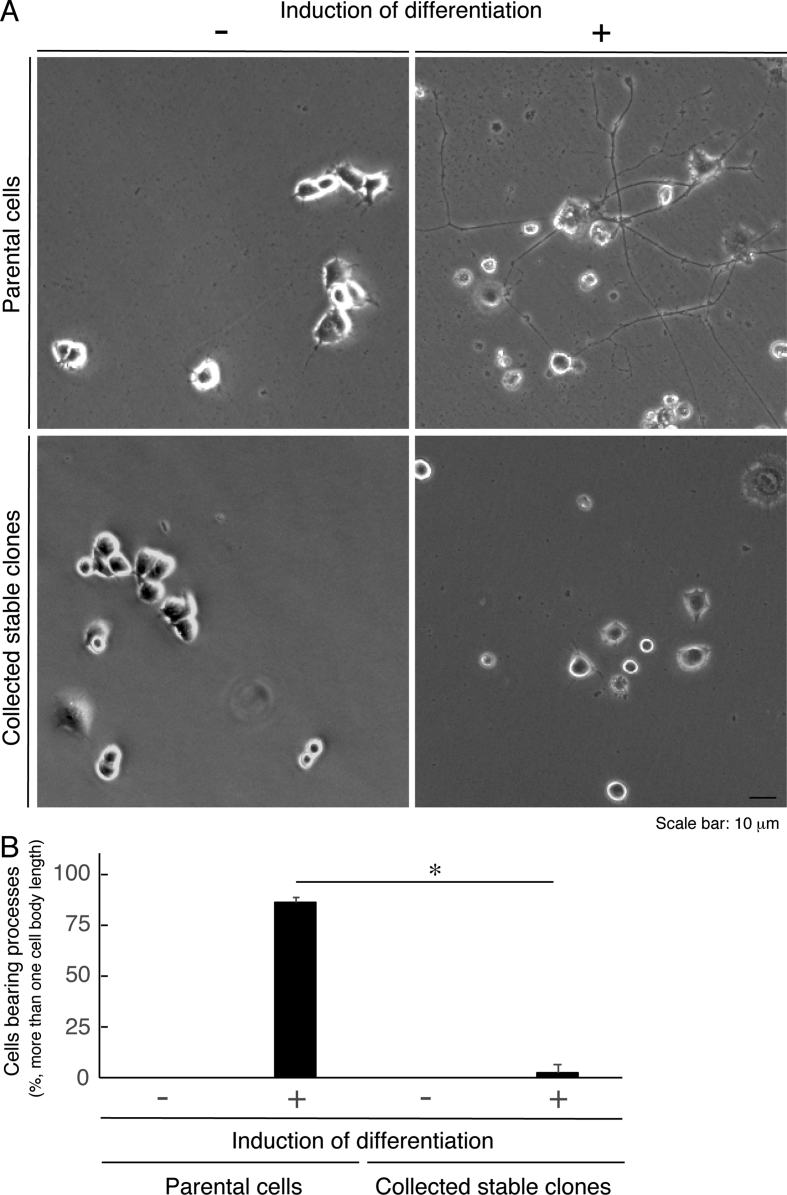
**Cells harboring AARS mutant constructs exhibit inhibitory differentiation.** (A) Parental N1E-115 cells or cells stably harboring the AARS mutant (Arg329-to-His) were allowed to differentiate for 5 days. (B) Cells with more than one-cell-body length process are considered to be harboring processes (differentiated cells) and are statistically shown (*, p < 0.01 of one-way ANOVA with post-hoc Fisher's test; n = 3 fields). Counted cell numbers were 198 and 212 in parental cells and stable clones, respectively. Left two bar graphs are from parental cells and right two ones are from cells stably harboring the AARS mutant.

The data shared in this article provide immunofluorescent and microscopic analyses of type 2N CMT disease-associated AARS mutant proteins (Arg329-to-His) for AARS protein localization and cellular differentiation. This position of the mutation in French family [1], [2], [3], [4] is different from the Asn71-to-Tyr mutation in Taiwanese family [5]. Fig. 1 describes cytoplasmic localization of GFP-tagged wild type AARS proteins and intracellular punctate localization of GFP-tagged AARS mutant proteins in COS-7 cells. In Fig. 2, Fig. 3, Fig. 4, GFP-tagged AARS mutant proteins are co-stained with antibodies against antigens of the endoplasmic reticulum (ER), Golgi body, and lysosome, respectively. Mutant proteins are partially co-localized with Golgi and lysosomal antigens (Fig. 3, Fig. 4). Additionally, parental neuronal N1E-115 cell line exhibits differentiated phenotypes with long processes whereas cells stably harboring mutant constructs exhibit decreased differentiated ones (Fig. 5).

## 2 Experimental design, materials and methods

### 2.1 Plasmid construction

Human AARS (GenBank Acc. No. NM_001605.2) was amplified from human corpus callosum cDNAs (Takara Bio, Shiga, Japan) and ligated into the GFP-expressing pEGFP-N3 vector (Takara Bio). The Arg329-to-His mutation (OMIN ID: 613287) was produced from pEGFP-N3-human AARS as the template, using the site-directed mutagenesis kit (TOYOBO Life Science, Osaka, Japan), according to the manufacturer's instructions. All DNA sequences were confirmed by sequencing (Fasmac, Kanagawa, Japan).

### 2.2 Cell culture, differentiation, and transfection

African green monkey epithelial-like COS-7cells (Human Health Science Research Resources Bank, Osaka, Japan) and mouse neuroblastoma N1E-115 cells (kindly provided by Dr. Daisuke Shiokawa, Tokyo Science University, Chiba, Japan) were cultured on 3.5-cm cell culture dishes (Greiner, Oberösterreich, Germany) with or without a coverslip in DMEM (Nakalai Tesque, Kyoto, Japan) containing 10% heat-inactivated FBS (Thermo Fisher Scientific, Waltham, MA, USA) and PenStrep (Thermo Fisher Scientific) in 5% CO_2_ at 37 °C. Cells were transfected with the plasmids using the ScreenFect A or ScreenFect A Plus transfection kit (FujiFilm, Tokyo, Japan), according to the manufacturers’ instruction. The medium was replaced 4h after transfection. Transfected cells were used for experiments 48h after transfection. To induce differentiation of N1E-115 cells, cells were cultured in DMEM containing PenStrep in 5% CO_2_ at 37 °C for 5 days. Cells harboring processes longer than one-cell-body length were estimated as differentiated cells [1].

### 2.3 Stable clone isolation

For isolation of N1E-115 cells stably harboring AARS (Arg329-to-His), cells were transfected with pEGFP-N3-AARS (Arg329-to-His). Growth medium containing 500 μg/ml G418 (Nacalai Tesque) was changed every 2 or 3 days, according to the manufacturer's instructions. After 14 days, G418-resistant colonies were collected and compared with phenotypes of their control parental cells.

### 2.4 Immunofluorescence

Cells on a coverslip were fixed with 4% paraformaldehyde (Nacalai Tesque) or 100% cold methanol (Nacalai Tesque). Cells were blocked with the Blocking One reagent (Nacalai Tesque) and incubated with primary antibodies (mouse monoclonal anti-ER antigen KDEL [MBL, Aichi, Japan] for the ER; mouse monoclonal anti-Golgi matrix protein of 130 kDa (GM130) [BD Biosciences, Franklin Lakes, NJ, USA] for the Golgi body; and mouse monoclonal anti-lysosomal-associated membrane protein 1 (LAMP1) [Abcam, Bristol, UK] for the lysosome) and, in turn, with Alexa Fluor-conjugated secondary antibodies (Thermo Fisher Scientific). The coverslips on the slide glass were mounted with the Vectashield reagent (Vector Laboratories, Burlingame, CA, USA) [6]. The fluorescent TIFF images were collected with a microscope system equipped with a laser-scanning Fluoview apparatus (Olympus, Tokyo, Japan) using Fluoview software (Olympus). Their resulting colored images were analyzed in the line plot analysis mode using the Image J software (URL: https://imagej.nih.gov/).

## References

[1] Y. Tatsumi, N. Matsumoto, N. Iibe, N. Watanabe, T. Torii, K. Sango, K. Homma, Y. Miyamoto, H. Sakagami, J. Yamauchi, CMT type 2N disease-associated AARS mutant inhibits neurite growth that can be reversed by valproic acid. Neurosci. Res. in press: DOI: 10.1016/j.neures.2018.09.016 10.1016/j.neures.2018.09.016 30261202

[2] Berger P., Niemann A., Suter U. (2006). Schwann cells and the pathogenesis of inherited motor and sensory neuropathies (Charcot-Marie-Tooth disease). Glia.

[3] Rossor A.M., Tomaselli P.J., Reilly M.M. (2016). Recent advances in the genetic neuropathies. Curr. Opin. Neurol..

[4] Latour P., Thauvin-Robinet C., Baudelet-Méry C., Soichot P., Cusin V., Faivre L., Locatelli M.C., Mayençon M., Sarcey A., Broussolle E., Camu W., David A., Rousson R. (2010). A major determinant for binding and aminoacylation of tRNA (Ala) in cytoplasmic alanyl-tRNA synthetase is mutated in dominant axonal Charcot-Marie-Tooth disease. Am. J. Hum. Genet..

[5] Lin K.P., Soong B.W., Yang C.C., Huang L.W., Chang M.H., Lee I.H., Antonellis A., Lee Y.C. (2011). The mutational spectrum in a cohort of Charcot-Marie-Tooth disease type 2 among the Han Chinese in Taiwan. PLoS One.

[6] Miyamoto Y., Torii T., Tago K., Tanoue A., Takashima S., Yamauchi J. (2018). BIG1/Arfgef1 and Arf1 regulate the initiation of myelination by Schwann cells in mice. Sci. Adv..

